# Trauma in Rapes and Assaults

**DOI:** 10.3390/children8121143

**Published:** 2021-12-06

**Authors:** Jean-Michel Darves-Bornoz

**Affiliations:** Centre Adolescents FSEF Henri-Danon-Boileau, University Hospital Bichat, 75018 Paris, France; jmdarvesbornoz@gmail.com

**Keywords:** trauma, adolescent, child, rape, incest, assault, maltreatment

## Abstract

Psychological trauma primarily affects children and adolescents; it mostly results from physical and sexual maltreatment. In the Medico-Judicial Unit Center for Sexual Violence Victims in Tours, France, which I joined in 1992 for research and to give treatment, underage patients represented about three-quarters of patients. At the same time, a national survey was conducted in collaboration with Marie Choquet’s “Adolescent Health” group (INSERM), which targeted several thousand adolescents representing the general population. It revealed that almost one out of five adolescents had experienced physical or sexual assault, and that although the number of sexual assaults probably does not exceed that of physical assaults, most of the time their psychological consequences do considerably exceed those of physical assaults. Several symptoms appear after experiencing rapes or assaults. They have a distinct semiology and independent evolutions. We isolated three of them: dissociative and phobic traumatic syndrome, re-experiencing traumatic syndrome, and borderline-like traumatic syndrome. They are generally triggered all at the same time or in close succession. Re-experiencing traumatic syndrome is profound, but the other two are often more worrying, particularly in relation to children and adolescents, because they generate disorders in their psychological development.

Psychological trauma in rapes and assaults is a serious public health issue. In France, the lack of epidemiological data on rapes and assaults led several research units to perform a series of collaborative studies from 1989 [1] onwards, such as those by Thérèse Lempérière [2,3,4], Jean-Pierre Lépine [5,6], and Marie Choquet [7,8]. Those studies were conducted in reference to the treatment and research program implemented in the *Adolescent Unit* at the *University Hospital* in Tours, which was followed by the *Psychological Trauma Victim Consultation,* which treated almost two hundred people a year (adults, adolescents, or children). For the most part, this research proceeded in the following chronological order: they started from observations among two categories of female inpatients suffering from severe psychiatric disorders—schizophrenia and manic-depressive bipolar disorder—for which sexual violence is very frequent and has harmful consequences. These observations were then replicated with psychiatric inpatients, no matter what disorder they had been diagnosed with; this eventually led to two systematic studies of incestuous or non-incestuous rape victims from the general population. The first one was cross-sectional, while the other was prospective and lasted over a year. Until now, no other epidemiological study has been conducted on this topic in France.

The purpose of this article is to provide a clinical approach to the aftermath of rapes and other physical maltreatments through an overview of decades of epidemiological and clinical research. While some patients recover quickly from trauma, most of them suffer from different forms of sequelae after these experiences, varying from subject to subject, especially according to their age and environment. This article will describe the distinct outcomes of these traumatic experiences.

## 1. History

Clinical psychiatry and psychotraumatology were born together in France. This is demonstrated by the categories of Briquet’s *Hystérie* [9], Charcot’s *Hystérie traumatique* [10], and Janet’s *Dissociation hystérique* [11]. These paved the way for Freud’s *Neurotica*; his theory states that the aetiology of *dissociative hysteria* lies in a traumatic occurrence [12], the nature of which he tried to specify not long after—“*a passive sexual experience before puberty: thus is the specific aetiology of hysteria*” [13]. The fact that Freud then disavowed this theory—due to lack of courage for some [14] or a denial of his own family history for others [15]—does not remove its relevance or continued presence within Freud’s ulterior theorisations up to the end of his life. Two elements remind us of the current lingering hesitation to highlight the realities and consequences of sexual crimes against children. Firstly, the fact that some circles within the medical community and society as a whole have resisted during the following sixty years. Secondly, that some of Freud’s close relations were zealous in burying this work within an uninteresting historical narrative (this was counterbalanced, admittedly, by relations who were just as close, and highly creative) [16,17,18,19]. Indeed, it is hard to imagine that a century had come to pass before such well-crafted work was examined again, even without hypothesising that the causes lay outside of the medical field. 

In order to explore this, let us recall what the state of knowledge was like at the time. In 1860, Tardieu published an article entitled *Etude médico-légale sur les sévices et mauvais traitements exercés sur des enfants* [20] in a scientific journal. Tardieu was a forensic medicine professor at the Université de Paris, the Dean of the Faculté de Médecine de Paris, and the President of the Académie de Médecine. The article documents thirty-two cases that he examined as an expert. His book *Etude médico-légale sur les attentats aux mœurs* was published as early as 1857. In the 1878 edition of this work, he explained that in France between 1858 and 1869, a total of 11,576 people had been accused of rape or had attempted rape. Among them, 9125 had been accused of raping or attempting to rape children. Tardieu assessed 616 of these cases; of them, 339 were rapes or attempted rapes on children under the age of 11. Tardieu’s successor, Professor Brouardel, specialised in the study of child rape. He gave conferences on the topic, and his book titled *Les attentats aux moeurs* was published within a collection of forensic medicine lectures from the Faculté de Médecine de Paris after he died [21]. The existence of severe maltreatment was therefore not foreign to the French medical community and social elites. Little by little, these experts’ opinions were patronisingly discredited for their supposed ingenuity in an area where allegedly nothing could be proved; subsequently, they were plainly rejected as the veracity of these allegations was challenged, and the concept of “pithiatism” was thereby promoted.

## 2. Nature of Traumatic Experiences

Traumatic assaults on the subjects that we are tasked with treating consist of physical or sexual violence; either with a mainly utilitarian goal (theft) or a perverse one (an assault with no other aim than itself); either in a professional context (armed assault, altercation at a shop counter, or behind a bus wheel) or a civil one (“suburban uprising”); either in an international setting (war) or a societal one (violent political confrontation); either on isolated subjects (rape) or on groups (hostages); and they can be further complicated by facts of another nature (traumatic loss and/or separation). As there is an intersubjectivity between a victim and an aggressor, these events stand out from other potentially traumatic situations, such as natural disasters for instance, in which traumatised subjects do not feel that they have been objectified by an aggressor through a sadistic relationship.

Psychological traumas predominantly involve children and adolescents; moreover, they mainly consist in physical and sexual maltreatment [22]. In the *Medico-Judicial Unit Center for Sexual Violence Victims* in Tours, about three-quarters of the patients are underage. A national survey was conducted among several thousand adolescents representing the general population, in collaboration with Marie Choquet’s “*Adolescent Health*” group (INSERM U472). It revealed that almost one out of five adolescents had experienced physical or sexual assaults, and that although the number of sexual assaults probably does not exceed that of physical assaults, most of the time their psychological consequences do considerably exceed those of physical assaults [23]. 

Indeed, not all of the various situations which are likely to generate traumas have the same intensity levels in their consequences, just as not all subjects have the same resistance levels. Moreover, the context or sequence in which the trauma occurs influences its psychopathological evolution. In particular, on top of the usual psychotraumatic clinical consequences, the chronic reiteration of trauma creates other specific clinical manifestations of its own. Therefore, suffering from a car accident but then being warmly supported by family or friends is not the same as suffering from war abuse for several years [24]; similarly, the wound caused by a single rape event endured by a thirty-year-old is not the same as the one caused by incest endured from one’s own father every day from six to sixteen years old [25]. With that in mind, knowing the differences between boys and girls regarding rapes and assaults is important. Boys who have been subjected to sexual assaults represent no less than a quarter of victims. These boys’ psychological distress is hardly less severe than that of girls. Moreover, distress expressed via deviant behaviours, which are a behavioural surfeit to psychological distress, are much more prevalent in boys than in girls [8].

Ultimately, like Edna Foa [26], we introduce rape as the first cause of psychological trauma, at least in peacetime, and maybe even in times of war, as rapes by mercenaries are as old as time. Our statement is based firstly on the numerous occurrences of this potentially traumatic wound; almost two million women have experienced rape in France, according to the INSERM team from Bicêtre [27], confirmed by the *Comité Français d’Education pour la Santé* [28]. It is also based on the fact that this event is highly trauma-inducing [22]. Indeed, re-experiencing traumatic syndrome chronically persists in most cases, and even more so if the rape was incestuous [24,25]. In fact, among psychiatric inpatients, when subjects suffer from a trauma history, most of the time it is a sexual one [5]. 

## 3. Trauma Clinics

Several psychotraumatic syndromes appear after experiencing rapes or assaults [29]. They each have a distinct semiology and independent evolution. We isolated three of them [5,29]: *dissociative and phobic traumatic syndrome, re-experiencing traumatic syndrome,* and *borderline-like traumatic syndrome*. They are generally triggered all at the same time or in close succession. Re-experiencing traumatic syndrome is profound; however, by many accounts, the other two are much more worrying, particularly in relation to children and adolescents, because they generate severe disorders in their psychological development [30].

*Dissociative and phobic traumatic syndrome* represents the way subjects defend themselves during the traumatic event and then attempt to treat both their identity wound and their re-experiences, respectively, by fragmenting their memory and psyche and by avoiding the world. What we call “dissociation” is a phenomenon that is both intra-psychological and somato-psychological [11]. Intra-psychological dissociation includes amnesia phenomena (about the traumatic event, or not), depersonalisation phenomena (for instance, with out-of-body experiences), derealisation phenomena (for instance, the feeling that they are watching their life as if it were a film on television), identity fragmentation phenomena (at worst, feeling like two different people), and automatisation phenomena (in particular, when running away from home). On various levels, these are responses to the pain of subjects who were unprepared for the emergence of a world-representation that contradicted the one they had until then. Somato-psychological dissociation in European psychiatric tradition, for example, a non-epileptic seizure or anaesthesia, means conversion. This responds to the pain that was also felt in the body, which used to be considered safe but has been proven not to be. It counteracts somatic re-experiencing [31]. Table 1 shows the strong link between traumatic experiences and psychological dissociations, conversions, and phobias. As for phobias, let us point out the most specific one, agoraphobia, and the least specific one, social anxiety disorder. Indeed, social anxiety disorders may come from traumatic consequences, as well as from complications due to a temperamentally low ability to avoid hostile situations. We sometimes notice that while the *re-experiencing syndrome* has disappeared, the only disorder that still remains consists of psychogenic amnesia or panic disorder with agoraphobia [32]. These results concur with the most recent theorisations of panic attacks. Indeed, in order to understand the first attack’s trigger, specialists need to include an environmental wound along with the other involved factors [33].

*Dissociative and phobic traumatic syndrome* is the first traumatic syndrome to appear after the traumatic event takes place. It can become chronic. In that case, everything seems to indicate that experimenting with this defence mechanism during the traumatic event leads to using the same mechanisms with a much higher frequency later, during other hostile but non-trauma-inducing events, when other people could have reacted, for example, with depression symptoms. Its persistence must come as a warning that *re-experiencing syndrome* may be present for a long period of time [29]. 

The clinical features of *re-experiencing traumatic syndrome* include reminiscences, nightmares, and trigger associations which set off a re-experience of the pain tied to the traumatic experience, or even the illusion of the traumatic experience itself. They are the most specific and sensitive out of all post-trauma symptom groups [22]. The American classification of mental disorders states that this syndrome is a necessary criterion for PTSD diagnosis but that it is not enough on its own. However, in practice, when we observe the presence of painful assault re-experiences, the other two symptom groups required for this category are rarely absent. Physically or sexually maltreated subjects prominently undergo painful re-experiences [24]. The diagnosis still applies to the majority of raped subjects one year after the traumatic event took place [6,32], and this chronologically constitutes one of the first therapeutic challenges at stake. Re-experiencing happens when an elementary sensory representation is recalled (for example, the image of the rapist’s eyes) through memories, nightmares, or trigger-associations (for example, a white car for someone who was assaulted inside a white car). It represents the traumatic event in its entirety and activates the emotions tied to it. Therefore, re-experiencing indicates a disordered representation of the past. 

In spite of how frequent it is among traumatised people (one out of two people are affected), highlighting their depression symptoms could lead to assumptions that treating such patients is not any more difficult than treating isolated depression. However, let us point out that the British psychiatry professor Sir David Goldberg, who dedicated his entire life to studying depression vulnerability factors, has no hesitation in claiming that sexual assaults are the first aetiological factors of depression [34]. 

*Borderline-like traumatic syndrome* can manifest in more psychological ways or more behavioural ways, depending on the cases or the moments in time. At first, “identity instability” is expressed on a psychological level and as a narcissistic depression (bad self-esteem, shame, guilt, abandonment disorder, feelings of emptiness, and the loss of vitality and identity) which can be so severe that it could evoke melancholy if its traumatic aetiology is not identified. The alteration of psychological development often complicates the identity disorder afterwards, sometimes quickly, particularly among children and adolescents. Indeed, an identity rebuilding process appears, with paranoid omnipotence fits or acting-out behaviours of various kinds. These well-documented borderline personality characteristics are rather typical of traumatic interactions between individuals [35,36]. This intersubjectivity, which promotes alienating identifications (to the aggressor among other things) and masochism often associated with traumatophilia, results in the alteration of relationships to other people and to the world. This disorder happens all the more frequently depending on how chronic and severe the trauma was. Thus, in Table 2, borderline-like characteristics are over-represented in incestuous rapes compared to non-incestuous rapes, in particular when it comes to bad self-esteem, abandonment disorder, feelings of emptiness, and a loss of vitality [25]. One should keep in mind that among those most severely traumatised, this syndrome is often the last one to keep resisting therapeutic measures, long after re-experiences have disappeared. This syndrome affects the subject’s expectations and ideals; therefore, it is unsurprising that out of the three syndromes, this one proves to be the most detrimental for children and adolescents. *Borderline-like traumatic syndrome* is a pathology that damages the representation of the future. 

## 4. Singularities

Some situations are characterised by a particular end to trauma. We have already mentioned the symptomatic differences between boys and girls [8,37] and the dissimilar prospects of incestuous rape victims as opposed to non-incestuous ones [25]. In this paragraph, we will only point out two lesser-known singularities, focusing on the psychological fate befalling rapes and assaults. 

### 4.1. Children Witnessing Their Mother’s Assault

Thanks to mental disorder classifications among other things, clinical researchers know about the specific semiologies of trauma re-experiences in children (for example, with games linked to part, or the entirety, of the traumatic experience). 

It is easy to investigate children’s trauma re-experiences verbally. Children will give an account of reminiscences or nightmares very simply, when prompted to do so. Most of the time, just like with adults, they would not spontaneously do so; therefore, stating the importance of verbal interaction during the child witnesses and their mother’s consultation does not have much inherent worth. 

Indeed, when a little girl who has been asked to represent the time frame when her mother was in danger—raped in her car under the threat of her children getting hurt—she will draw a car. We will use her drawing as a starting point to make associations, in front of her brother and mother, through our remark that a woman’s face seems to be surrounding the car. As for the little brother, after we requested for him to draw his family before difficulties started, he also represented a car. “This car belongs to everyone”, he says, which allows me to say something that is implicit for him but left unsaid between the two children and the mother. I tell him “that’s impossible, since your father and mother are separated”. In that way, two ideas that were being verbally avoided in this family, which has been reduced to the mother and her two children, are reintroduced in their three-way relationship—“*dad and mom are separated*”—as well as what was implicitly fantasised about—“*this wouldn’t have happened if my father had been there*”. 

Above all else, it is the children’s psychological development that is at stake after they witness rapes or assaults. That psychological dead end often leads to acting out according to various thought processes, for example, the birth of a fantasy or a thought such as “*the world is dangerous*” for phobic avoidance; “*I am worthless*” for suicide attempts, scarification, and self-harm; or “*never again will I surrender*” for tantrums from children identifying the aggressor. In the worst cases, masochism can be eroticised up to the point that they look for all sorts of potentially trauma-inducing situations (counter-phobic challenging behaviours and risk-taking). This is the reason why we stress the importance of looking for behavioural alerting in adolescents who have previously been involved in an assault; furthermore, their running away must always lead to an inspection for sexual assault histories [7,32,35].

Precocious affective development and a disproportionate investment in education can sometimes be observed. 

I had to take care of a four-year-old child who had witnessed his father’s attempt to murder his mother. When he came to my office with his mother, he always asked me for my keys and went to lock the door. So much so that when his mother one day asked me, “when will his disorders end?”, it seemed appropriate to answer, “when he will no longer ask for my keys in order to lock the office”. Indeed, by acting in such a way, this wary little boy for the first time tried to recreate the safe place that he had been lacking ever since the traumatic event, because he confusedly felt that he needed it in order to grow up. In school and at home, he was manifesting an irrepressible thirst for knowledge because his actual fantasy was: “*What will I do if my mother dies?*”. The behavioural response was characterised by learning a lot and quickly; growing up in order to be ready in case his mother died. 

The maniac defence from children attempting to resist their new world-representation, which they experience as hostile, with accelerated psychological development must not be related either to the diagnosis setting of real maniac conditions or to the diagnosis of attention-deficit disorder hyperactivity.

### 4.2. The Positive Ending of Trauma

*Borderline-like traumatic syndrome* exists along a continuum, with the severe end consisting of severe narcissistic depression, sometimes transforming into dangerous acts against oneself or others, and the benign end consisting of non-conformist behaviour. In those who have been able to perlaborate their narcissistic wound from a traumatic experience, atypicality and fearlessness to the world—which have become less phobogenic than before the traumatic experience—are common ways to move beyond trauma. Indeed, “healing” from trauma cannot happen within a conformist frame. In order to move beyond phobia, the subject has to go through contra-phobic defiance, which does not always worsen and turn into a characterised borderline pathology. Therefore, the subject’s evolution proves to be similar to that of the traumatised child’s, who wants to grow up quickly, or disproportionately thirsts for knowledge. This is why trauma partly works in favour of life: without contra-phobic defiance, we would all still be in our cribs. 

## 5. Conclusions

In summary, subjects whose trauma refers to an intersubjective relationship with others placed in the sadistic position most likely suffer from re-experiences, but also resort to intra- and somato-psychological dissociations and phobic avoidance of the world in order to prevent the pain brought about by panic attacks; at the same time, they also transform—in a harmful or benign way—their identity and narcissistic equilibrium. 

Rapes and assaults alter the usual way of representing the past. Indeed, with *re-experiencing traumatic syndrome,* the traumatic memory is reduced to proto-representation, for example, just the image of the rapist’s eyes; the pain experienced from the entire traumatic experience comes to be associated with this proto-representation. 

Rapes and assaults alter the usual way of representing the future. Indeed, with *borderline-like traumatic syndrome,* the wounded subject aims for a neo-identity, which implies new thought processes and new behavioural patterns in the future, for example, with an identification to the aggressor. 

The way in which a subject transitions from the first register to the second is determined by the defence mechanisms used in the traumatic experience, which we have gathered under the name of *dissociative and phobic traumatic syndrome*. Indeed, on the one hand, phobic avoidance revives the traumatic past day after day; on the other hand, phobia also brings a projection into the future: contra-phobic defiance. Similarly, above all else, both amnesia and conversion allow the subject to ignore the past, utilising personality fragmentation. Nevertheless, these dissociations also stimulate the emergence of neo-identities which are supported by new ideals and expectations for the future. With those, subjects attempt to adapt themselves to the world that has emerged after the trauma.

## Figures and Tables

**Table 1:** Links between constituted (*) psychiatric disorders and persistent traumatic re-experiencing—a prospective study of raped subjects over six months.

Traumatic Re-Experiencing			
	Still Re-Experiencing	No Re-Experiencing	Comparison
	at Six Months	at Six Months	of Both Groups
	%	%	*p*
Psychological dissociation	84	38	<0.0001
Conversions	75	42	<0.01
Agoraphobia	70	20	<0.0001
Simple phobia	56	25	<0.02
Social phobia	49	29	ns.
Panic disorder	18	0	<0.03
Depressions	53	8	<0.001
Sexual identity disorder	41	4	<0.001
Alcohol consumption excess	29	8	<0.05
Drug use	14	8	ns
Obsessional disorder	12	0	ns
Generalised anxiety	7	17	ns
Psychosis or bipolar disorder	7	13	ns
Anorexia or bulimia	20	8	ns

(*) The only results taken into account in this table are those of disorders that appeared precociously and persisted in one way or another during the six months duration [29].

**Table 2:** Illustration of the increased suffering of rape victims in cases of incest, through borderline-like psychological or behavioural features (*).

Rapes		
	Incestuous	Non-Incestuous
Frequent abandonment fear	64%	57%
Idealising of friends	28%	44%
Bad self-esteem	68%	37%
Running away from home impulsively	33%	21%
Suicide attempts	33%	26%
Emotional disorder of depressive nature	49%	31%
Lingering feeling of emptiness	76%	56%
Violence-inducing fits of anger	54%	42%
Dissociative incidents	84%	60%
At least five out of nine characteristics	58%	38%
Average number of characteristics	4.8	3.7

(*) The only results taken into account in this table are those of disorders that appeared precociously and persisted in one way or another during the six months duration [25].
